# Isolated Recurrence of Diffuse Large B-Cell Lymphoma Predominantly in the Iris and Ciliary Body

**DOI:** 10.1177/24741264241276602

**Published:** 2024-09-14

**Authors:** Amit V. Mishra, Shangjun Jiang, Matthew T.S. Tennant, Mark E. Seamone

**Affiliations:** 1 Alberta Retina Consultants, Edmonton, AB, Canada; 2 Department of Ophthalmology, University of Alberta, Edmonton, AB, Canada; 3 Section of Ophthalmology, Division of Surgery, University of Calgary, Calgary, AB, Canada

**Keywords:** lymphoma, iris lesion, intravitreal methotrexate, external beam radiation

## Abstract

**Purpose:** To describe a single case of systemic lymphoma recurring in the iris and ciliary body. **Methods:** A retrospective case review was performed. **Results:** A 75-year-old woman presented to the retina service with an iris mass in the left eye. Her medical history was significant for previous systemic diffuse large B-cell lymphoma treated with systemic chemotherapy. Aqueous sampling was significant for recurrence of the disease. Local therapy with intravitreal (IVT) methotrexate was initiated. Although there was initial improvement, an increased interval between injections led to disease recurrence. External beam radiation to the left eye was then applied, leading to a complete clinical remission. **Conclusions:** Systemic lymphoma presenting in the iris is a rare manifestation that should be considered on the differential for an amelanotic iris lesion. Although monotherapy with IVT methotrexate did not control the ocular disease in this patient, subsequent external beam radiation resulted in complete clinical remission.

## Introduction

Clinically detectable cases of systemic lymphoma with involvement of the iris are rare, with approximately 50 cases appearing in the PubMed database over the past 40 years.^1^ Although diffuse large B-cell lymphomas are the most common subtype of lymphoma (25% to 30% of non-Hodgkin lymphoma cases),^2^ fewer than 10 cases in which the iris was involved have been reported in the literature (Table 1).^3–7^ Systemic chemotherapy and radiation were used for treatment in those cases.

**Table 1.** Cases Reports of DLBCL Involving the Iris.

Author	Age (y)	Sex	Presenting Features	Treatment
Mashayehki et al^3^	51	M	Iris lesion, AC reaction	Chemotherapy
Mashayehki et al^3^	57	M	Iris lesion, AC reaction, glaucoma	None
Bawankar et al^4^	69	F	Hyphema, iris neovascularization, choroidal thickening, RD	Referred to hematology/radiation oncology; subsequently lost to follow-up
Ahmed et al^5^	59	M	Hypopyon, iris neovascularization	IVT bevacizumab, IVT MTX, systemic chemotherapy
Papaliodis et al^6^	74	M	Hypopyon	Radiation, systemic chemotherapy
Li et al^7^	50	F	Iris lesion, AC reaction, glaucoma, ciliary body thickening	None

Abbreviations: AC, anterior chamber; DLBCL, diffuse large B-cell lymphoma; IVT, intravitreal; MTX, methotrexate; RD, retinal detachment.

We report a case of recurrent diffuse large B-cell lymphoma in the iris that was initially treated with intravitreal (IVT) chemotherapy and subsequent radiation for persistent disease activity. To our knowledge, no other cases have been reported in which IVT chemotherapy was used as the initial treatment.

## Case Report

A 75-year-old woman with a history of treatment for diffuse large B-cell lymphoma was referred for evaluation of an iris lesion. She was initially treated with 6 cycles of chemotherapy comprising rituximab, cyclophosphamide, hydroxydaunomycin, vincristine, and prednisone for systemic disease.

A relapse occurred with secondary central nervous system involvement, and the patient was treated with salvage chemotherapy comprising 1 cycle of rituximab, etoposide, methylprednisolone, high-dose cytarabine, and cisplatin and 1 cycle of rituximab, dexamethasone, cytarabine, and cisplatin. Mobilization of the peripheral blood stem cells was performed after chemotherapy to ensure an adequate number of stem cells for transplantation. Autologous stem cell transplantation resulted in a good metabolic response.

**Figure 1.**
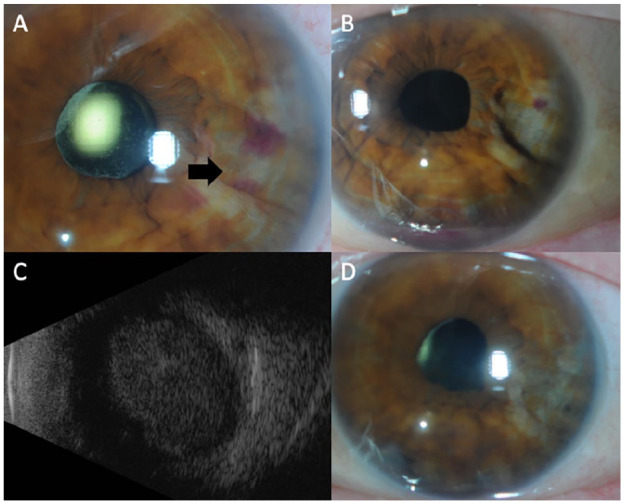
(A) Anterior segment photograph of the left eye shows a temporal iris mass with central hemorrhage overlying the lesion (arrow). (B) Remission of the iris mass with cavitation of the iris after intravitreal methotrexate therapy with evidence of a small hyphema. (C) B-scan ultrasound of the choroidal mass at the time of lymphoma recurrence; longitudinal scan at 6 o’clock. (D) Resolution of the iris mass after external beam radiation therapy.

**Figure 2.**
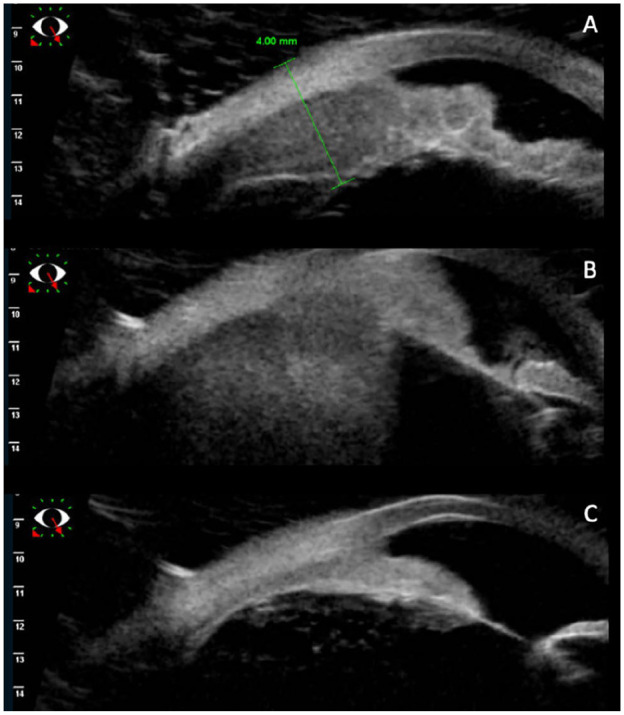
(A) Ultrasound biomicroscopy shows lymphoma involvement of the iris and ciliary body on presentation. (B) Progression of the iris lymphoma into the ciliary body at the time of lymphoma recurrence. (C) Resolution of the iris mass after external beam radiation therapy.

Nine months after autologous stem cell transplantation, the patient was evaluated in the retina clinic. The visual acuity (VA) was 20/20 OD and 20/25 OS. A temporal iris mass was noted in the left eye with associated iris thickening (Figures 1A and 2A). A layered hyphema and white hypopyon (candy-cane hyphema) were present with 3+ cells in the anterior chamber. The vitreous was significant for 2+ cells and haze. Uveal involvement including the ciliary body was seen on imaging (Figure 2A). There was no posterior pole choroidal thickening on optical coherence tomography.

An anterior chamber washout was performed, and aqueous fluid was sent for cytology. The results showed CD5-negative/CD10-negative large B-cell lymphoproliferation with CD43 positivity. CD19-positive B cells comprised 92% of the lymphoid subset. A positron emission tomography/computed tomography scan was performed, showing no evidence of disease outside the eye. Magnetic resonance imaging of the brain showed subtle thickening in the soft tissue of the anterior left globe.

A discussion with the oncology team led to the decision to begin with local therapy and to consider radiation if the response was minimal. The initial plan was to start combined therapy with IVT methotrexate and IVT rituximab. Because of a lack of insurance coverage, rituximab was not available; therefore, methotrexate monotherapy was initiated. Twice weekly therapy consisting of IVT methotrexate (400 mg/0.1 mL) was started. After 2 weeks, the injection interval was extended to once weekly for 4 treatments.

There was an initial reduction in tumor size with treatment (Figure 1B). The interval between injections was then extended to every 2 weeks, resulting in a new hyphema and a VA of 20/60. Ultrasound biomicroscopy showed enlargement of the ciliary body, which suggested extension of disease (Figure 2B), while a posterior examination showed an inferior choroidal mass (Figure 1C). The patient was referred to the radiation oncology service for external beam radiation (2000 cGY dose over 10 fractions) applied to the whole eye. After treatment, involution of the intraocular mass occurred (Figures 1D and 2C).

At 5 months, there was no evidence of ocular recurrence and the VA remained stable at 20/60. Unfortunately, there was further relapse of diffuse large B-cell lymphoma in the left thigh, and palliative treatment was commenced. The patient died 10 months after initially presenting to the retina clinic.

## Conclusions

Iris lymphomas typically present with clinical signs and symptoms that may overlap with those of anterior uveitis.^1,3^ Other considerations on the differential include presumed tubercular or sarcoid granuloma.^8,9^ Features such as hyphema, abnormal iris vessels, and visible lymphoid infiltration of the conjunctiva, iris, or choroid can help differentiate iris lymphoma from anterior uveitis; however, a definitive diagnosis cannot be made without tissue sampling or aqueous cytology.^1,3^ Ultrasound biomicroscopy is valuable in identifying the extent of iris and ciliary body infiltration, while B-scan imaging can characterize choroidal thickening and is especially helpful when there is media opacity.

Once the aqueous fluid or tissue sample has been obtained, the diagnosis of iris lymphoma is confirmed based on cytomorphology and ancillary testing that includes flow cytometry, immunohistochemistry, and cytogenetics.^10^ The differential diagnoses for iris lymphoma include benign reactive lymphoid hyperplasia, amelanotic iris nevi, amelanotic iris melanoma, juvenile xanthogranuloma, and autoimmune uveitis in the setting of autologous bone marrow transplantation.^1^

In our case, the treatment options, which included initiation of IVT therapy with the option of adjunct radiation if required, were discussed with the patient. IVT therapy and radiation were both considered because of the localized area of recurrence. There had been no recurrence of lymphoma outside the left eye when the patient presented to the retina clinic. Fortunately, lymphoma is rather radiosensitive; however, most institutions would agree that low-grade extranodal marginal zone lymphoma is treated at a lower dose (20 to 24 Gy) while high-grade lymphoma (eg, diffuse large B-cell lymphoma) is treated at a higher dose (30 Gy).^11,12^ In cases of palliative radiation, lower doses are often used. This knowledge is derived from the treatment of choroidal lymphoma, which tends to be low grade.

Although our patient had a good initial response to IVT methotrexate, there was progression of disease involving the iris and ciliary body in addition to choroid involvement after the treatment interval was extended to every 2 weeks. External beam radiation therapy with a mean dose of 28.67 Gy for high-grade secondary choroidal lymphoma has been shown to be beneficial, achieving long-term regression in 96% of cases.^11^ Previous case reports of iris and ciliary body lymphomas also show good outcomes after external beam radiation therapy, with no ocular recurrence,^3^ as seen in our patient.

## References

[1] Kakkassery V Coupland SE Heindl LM . Iris lymphoma-a systematic guide for diagnosis and treatment. Surv Ophthalmol. 2021; 66(1):41-53. doi:10.1016/j.survophthal.2020.06.003 32585164

[2] Padala SA Kallam A . Diffuse large B cell lymphoma. 2022. Accessed February 1, 2023. http://www.ncbi.nlm.nih.gov/pubmed/25805585 32491728

[3] Mashayekhi A Shields CL Shields JA . Iris involvement by lymphoma: a review of 13 cases. Clin Exp Ophthalmol. 2013;41(1):19-26. doi:10.1111/j.1442-9071.2012.02811.x 22594613

[4] Bawankar P Das D Bhattacharjee H , et al. Systemic diffuse large B-cell lymphoma masquerading as neovascular glaucoma. Indian J Ophthalmol. 2018;66(2):317-319. doi:10.4103/ijo.IJO_746_17 29380792 PMC5819129

[5] Ahmed H James A Enghelberg M . Successful use of intravitreal bevacizumab and methotrexate in a case of neovascularization of the iris and pseudohypopyon secondary to recurrent diffuse large B-cell lymphoma. Cureus. 2022;14(2):e22578. doi:10.7759/cureus.22578 PMC8958145 35371675

[6] Papaliodis GN Montezuma SR . Pseudo-hypopyon as the presenting feature of recurrent B-cell lymphoma. Ocul Immunol Inflamm. 2008;16(3):121-122. doi:10.1080/09273940802026052 18569803

[7] Li Z Lin Z Zhong Y Shen X . Iris metastasis of diffuse large B-cell lymphoma misdiagnosed as primary angle-closure glaucoma: a case report and review of the literature. Open life Sci. 2021;16(1):61-68. doi:10.1515/biol-2021-0008 33817299 PMC7874580

[8] Murthy SI Rathi VM Tyagi M Mishra DK Pappuru RR . Presumed intraocular tuberculosis manifesting as unilateral iris granuloma. Ocul Immunol Inflamm. 2020;28(7):1056-1059. doi:10.1080/09273948.2019.1699578 31944133

[9] Rejdak R Pogorelov P Mardin CY Szkaradek M Juenemann AGM . Solitary sarcoid granuloma of the iris mimicking tuberculosis: a case report. J Ophthalmol. 2014;2014:656042. doi:10.1155/2014/656042 PMC3964902 24734169

[10] Coupland SE Damato B . Lymphomas involving the eye and the ocular adnexa. Curr Opin Ophthalmol. 2006;17(6):523-531. doi:10.1097/ICU.0b013e328010948d 17065920

[11] Mashayekhi A Hasanreisoglu M Shields CL Shields JA . External beam radiation for choroidal lymphoma: efficacy and complications. Retina. 2016;36(10):2006-2012. doi:10.1097/IAE.0000000000001026 27031528

[12] Zimmermann M Oehler C Mey U Ghadjar P Zwahlen DR . Radiotherapy for non-Hodgkin’s lymphoma: still standard practice and not an outdated treatment option. Radiat Oncol. 2016;11(1):110. doi:10.1186/s13014-016-0690-y 27577712 PMC5004297

